# Contribution of the Infection-Associated Complement Regulator-Acquiring Surface Protein 4 (ErpC) to Complement Resistance of *Borrelia burgdorferi*

**DOI:** 10.1155/2012/349657

**Published:** 2012-01-30

**Authors:** Claudia Hammerschmidt, Teresia Hallström, Christine Skerka, Reinhard Wallich, Brian Stevenson, Peter F. Zipfel, Peter Kraiczy

**Affiliations:** ^1^Institute of Medical Microbiology and Infection Control, Frankfurt University Hospital, Paul-Ehrlich-Stra*β*e 40, 60596 Frankfurt, Germany; ^2^Department of Infection Biology, Leibniz Institute for Natural Products Research and Infection Biology, Beutenbergstra*β*e 11a, 07745 Jena, Germany; ^3^Institute of Immunology, University of Heidelberg, Im Neuenheimer Feld 305, 69120 Heidelberg, Germany; ^4^Department of Microbiology, Immunology and Molecular Genetics, University of Kentucky College of Medicine, Lexington, KY 40536, USA; ^5^Friedrich Schiller University of Jena, 07737 Jena, Germany

## Abstract

*Borrelia burgdorferi* evades complement-mediated killing by interacting with complement regulators through distinct complement regulator-acquiring surface proteins (CRASPs). Here, we extend our analyses to the contribution of CRASP-4 in mediating complement resistance of *B. burgdorferi* and its interaction with human complement regulators. CRASP-4 (also known as ErpC) was immobilized onto magnetic beads and used to capture proteins from human serum. Following Western blotting, factor H (CFH), CFH-related protein 1 (CFHR1), CFHR2, and CFHR5 were identified as ligands of CRASP-4. To analyze the impact of native CRASP-4 on mediating survival of serum-sensitive cells in human serum, a *B. garinii* strain was generated that ectopically expresses CRASP-4. CRASP-4-producing bacteria bound CFHR1, CFHR2, and CFHR5 but not CFH. In addition, transformed spirochetes deposited significant amounts of lethal complement components on their surface and were susceptible to human serum, thus indicating that CRASP-4 plays a subordinate role in complement resistance of *B. burgdorferi*.

## 1. Introduction

Lyme borreliosis, caused by spirochetes of the *Borrelia burgdorferi* sensu lato complex, is the most prevalent vector-borne anthropozoonosis in Eurasia and the United States [1]. The ability of spirochetes to perpetuate their natural vertebrate-tick infectious cycle spirochetes requires an array of mechanisms to successfully colonize their tick vectors and rodent reservoir hosts, survive in diverse environments, and evade host innate and adaptive immune responses. Recently, it has been shown that certain genospecies resist complement-mediated killing of human serum, in particular *B. burgdorferi* sensu stricto (hereafter referred to as *B. burgdorferi*), *B. afzelii*,* B. spielmanii*, and *B. bavariensis* (formerly known as *B. garinii* OspA serotype 4 strains) [2–5]. Elucidation of the underlying molecular mechanism(s) of complement resistance among Lyme disease spirochetes revealed that binding of the host complement regulators factor H (CFH) and factor H-like protein 1 (FHL1) to the bacterial surface directly correlates with serum resistance [3, 6–10]. In contrast, *B. garinii*, *B. valaisiana*, and *B. lusitaniae* are highly susceptible to complement-mediated killing and either do not bind, or bind inadequate levels of complement regulators [2, 4, 10–12]. 

Complement plays a central role in the recognition and elimination of invading microorganisms [13]. Upon activation of the initial steps of the complement cascade via the classical, alternative, or lectin pathway, a C3 convertase is generated which cleaves the central component C3 into its reactive fragments C3a and C3b. The highly reactive C3b fragment covalently binds to molecules, proteins, and nearby membranes, thereby leading to opsonization of the intruding microorganisms. This initial step is necessary for clearance of foreign microorganisms by phagocytosis, formation of the C3 convertase, and assembly of both the C5 convertase and the membrane attack complex (MAC). To protect host cell surfaces from uncontrolled and continuous activation, the complement system is well balanced and finely tuned by diverse fluid phase and membrane-anchored negative regulators [14–16]. CFH and FHL1 are the key fluid phase regulators of the human alternative pathway and act as cofactors for factor-I-mediated inactivation of C3b to iC3b, compete with factor B for binding to C3b, and finally support the dissociation (decay-accelerating activity) of the alternative pathway C3 convertase, C3bBb [16–20]. CFH is composed of 20 individually folding protein domains termed short consensus repeats (SCRs) of which the four N-terminal-located SCRs exhibit the complement regulatory activity. FHL1 is a 42 kDa glycoprotein, comprised of the seven amino-terminal SCRs of CFH plus four unique amino acids at the C-terminus [17, 20]. The human CFH family includes additional “factor H-related” proteins (CFHR), namely, CFHR1, CFHR2, CFHR3, CFHR4A, CFHR4B and CFHR5, all of which are encoded by distinct genes located in the regulators of complement activation (RCA) gene cluster on human chromosome 1 [21–23]. The C-terminal SCR domains of the CFHR proteins share high degrees of similarity to the C-terminal surface binding region of CFH, that is, SCRs 18–20 [16, 24]. The CFHR1 protein consists of five SCRs and exists in two glycosylated forms, the 37 kDa CFHR1*α* protein with one and the 43 kDa CFHR1*β* protein with two carbohydrate chains attached [25, 26]. CFHR1 is a complement regulator that blocks C5 convertase activity as well as assembly and membrane insertion of the terminal membrane attack complex [27]. CFHR2 is composed of four SCRs and is found in plasma as a nonglycosylated 24 kDa form (CFHR2) and a glycosylated 29 kDa form (CFHR2*α*) [28]. The function(s) of CFHR2 is as still unclear. The 65 kDa CFHR5 protein is comprised of 9 SCRs and displays cofactor activity for factor-I-mediated inactivation of C3b [29, 30]. CFHR5 also inhibits the activity of the fluid phase C3 convertase. 

Lyme disease *Borreliae* camouflage themselves with host-derived complement regulators through three groups of genetically unrelated genes/proteins collectively termed complement regulator-*a*cquiring *s*urface *p*roteins or “CRASPs” [3, 9, 31–35]. All investigated serum-resistant borrelial strains so far express the CRASP-1 protein in different combinations with CRASP-2, CRASP-3, CRASP-4, and/or CRASP-5. Based on the binding profile for complement regulators, CRASPs expressed by *B. burgdorferi* are divided into CFH and FHL1 binding proteins that do not bind CFHR1 (CRASP-1/CspA and CRASP-2/CspZ) and molecules that interact with CFH and CFHRs, but not FHL1 (CRASP-3/ErpP, and CRASP-4/ErpC, CRASP-5/ErpA) [9, 34, 36–39]. The potential of single CRASP-molecules in mediating complement resistance of *B. burgdorferi* s.s. is still under debate. Borrelial strains lacking functional CRASP-1 and CRASP-2 are highly susceptible to complement-mediated killing, and complementation with the respective CRASP encoding genes restores the serum-resistant phenotype [31, 40–42]. The contributions of the CFH and CFHR-binding CRASP-3 and CRASP-5 proteins in facilitating complement resistance of *Borreliae* are disputed. Heterologous production of either CRASP-3 or CRASP-5 in a *B. garinii *strain lacking all functional CRASP molecules failed to convert the serum-sensitive phenotype of the wild-type strain [39]. In contrast, Kenedy and Akins have shown that CRASP-3 and CRASP-5 produced in a CRASP-1 deletion strain lead to increased survival in human serum as compared to a serum-sensitive strain lacking CRASP-1 [43].

In the present studies, we extended our previous investigations on the CFH- and CFHR1 binding capacity of CRASP-4/ErpC protein to additional proteins derived from human serum and their contributions to convey complement resistance. To this end, a *B. garinii* strain that ectopically produced CRASP-4 was generated by transformation with a shuttle vector harboring the CRASP-4 encoding *erpC* gene, then the transformed strain was assayed for (i) the ability to bind human complement regulators, (ii) surface deposition of complement activation products, and (iii) survival in human serum. Using recombinant CRASP-4, two additional members of the human CFH protein family, CFHR2 and CFHR5, were identified as novel ligands for CRASP-4 of *B. burgdorferi* whereby CFHR2 showed stronger binding capacity for CRASP-4 as compared to CFHR1 and CFHR5. However, borrelial cells producing CRASP-4 on their surface did not bind CFH. Upon incubation in human serum, large amounts of activated complement components were deposited onto the surfaces of CRASP-4 producing cells and the bacteria did not survive. This suggests that binding of CFHR1, CFHR2, and CFHR5 is not sufficient to protect spirochetes from complement-mediated bacteriolysis once complement is activated.

## 2. Material and Methods

### 2.1. Bacterial Strains and Culture Conditions

*B. burgdorferi* strains LW2 (skin isolate, Germany), *B. garinii *isolate G1 (CSF isolate, Germany), *B. garinii* transformants G1/pKFSS1 as well as G1/pCRASP-4 were grown at 33°C for 2 to 4 days to midexponential phase (1 × 10^7^ to 5 × 10^7^ spirochetes/mL) as described previously [39]. *Escherichia coli* DH5*α* used for cloning experiments and protein expression was grown at 37°C in yeast tryptone broth, supplemented with the appropriate antibiotics.

### 2.2. Human Sera and Polyclonal and Monoclonal Antibodies

Normal human serum (NHS) obtained from 20 healthy human blood donors without known history of spirochetal infections was used as a source of complement regulators. The study and the respective consent documents were approved by the ethics committee at the Goethe University of Frankfurt (control number 160/10). All blood donors provided written, informed consent.

A polyclonal anti-CFH antiserum was utilized to detect human CFH, CFHR1, and CFHR2 (Merck Biosciences, Bad Soden, Germany and Complement Technology, Tyler, TE). Rabbit polyclonal anti-CFHR1 antibody or monoclonal antibody JHD 7.10 was used for detection of CFHR1 and CFHR2 and CFHR5 [39]. The goat anti-human C3 and C6 antibodies were purchased from Calbiochem, and the monoclonal anti-human C5b-9 antibody recognizing the MAC was obtained from Quidel (San Diego, CA). MAb L41 1C11 was used to detect the periplasmic FlaB protein. For analyzing surface-exposed CRASP-4, a rabbit polyclonal antiserum that recognizes CRASP-4 and CRASP-5 was used [44, 45].

### 2.3. Expression of Recombinant CFHR1, CFHR2, and CFHR5

Recombinant CFHR1 was expressed in *Spodoptera frugiperda *Sf9 insect cells infected with recombinant baculovirus. The cloning of various deletion constructs, expression, and purification have been described previously [38].

The full length CFHR2 cDNA was cloned into pPICZ*α*B (Invitrogen), and the protein was expressed in the yeast *Pichia pastoris* strain X33 according to standard protocols [39]. The full length CFHR5 cDNA was cloned into pBSV-8His and expressed in the baculovirus system as described [46]. All expressed His-tagged recombinant proteins were purified by Ni^2+^ chelate affinity chromatography as described [46].

### 2.4. Expression of Recombinant CRASP-4

The construction of vector pBLS528 used for the production of amino-terminally polyhistidine-tagged CRASP-4 (ErpC) was described previously [47]. The *erpC *encoding sequence of *B. burgdorferi *strain LW2 is identical to the sequence of the *erpC *gene of *B. burgdorferi *type strain B31. 

Expression of recombinant CRASP-4 protein was induced in DH5*α* at an OD_600_ of 0.6 by the addition of 0.2 mM IPTG. Following incubation for 4 h at room temperature, cells were centrifuged (5000 g, 20 min, 4°C) and subsequently suspended in lysis buffer (300 mM NaCl, 56 mM NaH_2_PO_4_ pH 8, and 10 mM Imidazole) containing 50 mg/mL lysozyme. Bacterial cells were lysed by 6 rounds of sonication for 30 sec using a Branson B-12 sonifier (Heinemann, Schwäbisch Gmünd, Germany). After centrifugation (14000 g, 20 min, 4°C), supernatants were filtered through 0,45 *μ*m filters and stored at −20°C for later purification via affinity chromatography.

### 2.5. Serum Incubation with Magnetic Beads Coated with His-Tagged CRASP-4 Protein

Purified CRASP-4 (20 *μ*g) was incubated with 50 *μ*L of magnetic beads (Dynabeads TALON, Invitrogen Dynal AS, Oslo, Norway) for 10 min at room temperature as recommended by the manufacturer. After four wash steps with phosphate buffer (50 mM phosphate, 300 mM NaCl, 0.01% Tween 20), histidine-tagged proteins coupled onto beads were incubated with NHS for 1 h on ice. After extensive washing with phosphate buffer, bound proteins were eluted with 50 *μ*L of 100 mM glycine-HCl (pH 2.0) for 15 min. The eluate and the last wash fraction were separated by 12.5% SDS-PAGE under nonreducing conditions followed by silver staining.

### 2.6. Construction of Shuttle Vectors

To allow ectopic expression of CRASP-4 by the serum-sensitive *B. garinii *strain G1, a shuttle vector was generated by using plasmid pKFSS1, a streptomycin-resistant derivative of pBSV2 [48]. The CRASP-4 encoding *erpC* gene plus its native promotor region was amplified from *B. burgdorferi* strain LW2 by PCR using primers containing the respective restriction sites and then sequenced (Table 1). The sequence of the *erpC* gene of *B. burgdorferi *strain LW2 is identical to that of *B. burgdorferi* type strain B31. Amplicons were hydrolyzed with HindIII and subsequently cloned into pKFSS1 at the corresponding restriction site, yielding shuttle vector pCRASP-4. The inserted sequence was subjected to nucleotide sequencing to verify that no mutations had been introduced during PCR and cloning procedures.

### 2.7. Transformation of Serum-Sensitive *B. garinii*

The noninfectious, serum-sensitive* B. garinii* strain G1 was grown in BSK medium and harvested at midexponential phase (5 × 10^7^ to 1 × 10^8^ cells/mL). Electrocompetent cells were prepared and transformed as described previously [39]. For selection of transformants, cells were diluted into 100 ml BSK medium containing 20 *μ*g/mL streptomycin, then 200 *μ*L aliquots were transferred into 96-well plates (Corning). After four to six weeks of incubation at 33°C, wells were evaluated for growth by color change of the medium and by dark-field microscopy for the presence of motile spirochetes. Several clones were expanded in 1 mL fresh BSK medium containing streptomycin (20 *μ*g/mL) for 7 to 14 days. Transformed bacteria were then maintained in BSK medium containing 20 *μ*g/mL streptomycin.

### 2.8. PCR Analysis of Transformed Borrelial Cells

Streptomycin-resistant clones of transformed *B. garinii* were characterized by PCR amplification of the introduced *erpC* gene and the recombinant plasmids streptomycin resistance gene (*aadA*) using specific primers (Table 1). The native *B. garinii flaB* gene was also amplified via PCR as a positive control. Ten microliter aliquots of bacterial cultures grown to midexponential phase were used for direct PCR. PCR was carried out for 25 cycles using the following parameters: denaturation at 94°C for 1 min, annealing at 50°C for 1 min, and extension at 72°C for 1 min. Reaction products were separated by agarose gel electrophoresis, and DNA was visualized by ethidium bromide staining and ultraviolet light.

### 2.9. SDS-PAGE, Western Blot, and Ligand Affinity Blot Analysis

Bacterial cell lysates were subjected to 10% Tris/Tricine-SDS-PAGE under reducing conditions and samples obtained by serum adsorption (last wash and eluate fractions) were separated by SDS-PAGE under nonreducing conditions as previously described [34]. 

For ligand affinity blot analysis, membranes were incubated for 1 h with normal human serum. After four washings with TBS containing 0,2% Tween20, membranes were incubated for 1 h with either a polyclonal goat CFH antiserum, polyclonal anti-CFHR1 antiserum that recognizes CFHR1, CFHR2, and CFHR5 and CFH or mAb JHD 7.10 which recognizes all three CFHRs but not CFH [38, 39, 49]. Following four washings with TBS containing 0,2% Tween 20, membranes were incubated with an appropriate peroxidase-conjugated secondary antibody for 1 h. Detection of bound proteins was performed using 3,3',5,5'-tetramethylbenzidine (TMB) as a substrate. 

For Western blot analysis, membranes were incubated for 1 h at room temperature with antisera recognizing CRASP-4/ErpC and CRASP-5/ErpA (*α*CRASP-4), CFH, CFHR1, or FlaB (L41 1C11). Following four wash steps with TBS containing 0,2% Tween20, membranes were probed with appropriate peroxidase-conjugated secondary antisera (Dako, Glostrup, Denmark) for 60 min at room temperature and bound antibodies were detected using TMB.

### 2.10. ELISA

Microtiter plates (Nunc-Immuno Module) were coated with CRASP-4 (5 *μ*g/mL) over night at 4°C. Microtiter plates were washed with PBS containing 0.1% Tween 20 and treated for 1 h at RT with blocking buffer (AppliChem GmBH, Darmstadt, Germany). After washing, equimolar amounts (33 *μ*M) of CFH, CFHR1, CFHR2, or CFHR5 were added and incubated for 1 h at RT. Thereafter, the wells were washed and bound CFH or CFHR proteins were detected with either goat CFH polyclonal antiserum or MAb JHD 7.10, which reacts with all three CFHRs [39, 49]. After washing, bound proteins were identified using appropriate secondary horseradish peroxidise-coupled antisera. Detection was performed with 1,2-phenylenediamine dihydrochloride as a substrate (OPD, DakoCytomation, Glostrup, Denmark) and absorbance was measured at 490 nm.

### 2.11. In Situ Protease Accessibility Experiments

Viable *Borreliae* were gently washed and resuspended in 500 *μ*L PBS to obtain a density of 8 × 10^5^/*μ*L. Subsequently, proteinase K and trypsin (Sigma-Aldrich, Deisenhofen, Germany) were separately added to a final concentration of 25 and 100 *μ*g/mL, respectively. Intact spirochetes without protease treatment served as a control. Following incubation for 2 h at room temperature, proteinase K and trypsin were inactivated by addition of phenylmethylsulfonyl fluoride (Sigma-Aldrich) (50 mg/mL in isopropanol). Cells were then washed gently twice with PBS-5 mM MgCl, resuspended in 20 *μ*L of the same buffer, then lysed by sonication 5 times for 30 sec using a Branson B-12 sonifier (Heinemann, Schwäbisch Gmünd, Germany). Aliquots were separated using Tris/Tricine-SDS-PAGE as described above.

### 2.12. Serum Adsorption Assay

To assess binding of serum proteins to viable borrelial cells, a serum adsorption assay was employed as described previously [7, 50]. Briefly, borrelial cells (1 × 10^9^ cells) grown to midexponential phase were washed and subsequently resuspended in 750 *μ*L NHS supplemented with 34 mM EDTA (pH 8.0) to avoid complement activation. After 1 h incubation and four washes with PBS containing 0.01% Tween 20, proteins bound to the cells surface were eluted with 100 mM glycine-HCl (pH 2.0) for 15 min. Cells were removed by centrifugation at 14000 g for 10 min at 4°C, and the supernatant and the last wash were separated by SDS-PAGE under nonreducing conditions and analyzed by Western blotting as described above.

### 2.13. Serum Susceptibility Testing

Serum susceptibility of *B. garinii* isolate G1, G1/pKFSS1, and G1/pCRASP-4 was assessed by a growth inhibition assay as described previously [3, 42]. Briefly, aliquots (1.25 × 10^7^ cells) of highly motile spirochetes were diluted into final volumes of 100 *μ*L fresh BSK medium, which contains 240 *μ*g/mL phenol red. As bacteria grow in BSK, the medium acidifies and the pH indicator dye turns from red to yellow. One hundred microliters of NHS or 100 *μ*L heat-inactivated NHS was added to each aliquot of bacteria. Bacteria were then incubated in 96-well microtiter plates for 8 days at 33°C. For controls, aliquots of bacteria were also incubated with 100 *μ*L BSK medium instead of NHS. Bacterial growth was monitored daily by measuring the ratio of culture medium absorbance at 562 versus 630 nm, using an ELISA reader (PowerWave HT; Bio-Tek Instruments, Winooski, VT). For calculation of the growth curves the Gen5 software (Bio-Tek Instruments, Winooski, VT) was used. Each experiment was conducted at least three times, and means ± SD were calculated.

### 2.14. Immunofluorescence Assay

Spirochetes grown to midexponential phase were harvested by centrifugation (5000 g, 30 min), washed, and resuspended in veronal buffered saline (VBS, supplemented with 1 mM Mg^2+^, 0.15 mM Ca^2+^, and 0.1% gelatin, pH 7.4). 

For detection of deposited complement components on the bacterial surface, spirochetes (6 × 10^6^) were incubated in 25% NHS and, as a control, in 25% heat-inactivated NHS for 30 min at 37°C with gentle agitation as previously described [3, 42]. 

In order to detect surface-exposed proteins, polyclonal rabbit anti-CRASP-4 antiserum (1:50 dilution) was added to the cells for 1h at 37°C with gentle agitation. After two washes with PBS containing 1% BSA, 10 *μ*L aliquots of the cell suspensions were spotted on glass slides and allowed to air-dry overnight (= unfixed cells). Slides were then fixed in methanol for 10 min and air-dried for 1 h, followed by incubation with an adequate Alexa 488-conjugated antibody. Slides were then gently washed four times with PBS and mounted on ProLong Gold antifade reagent (Molecular Probes) containing DAPI before being sealed. Slides were visualized at a magnification of ×1,000 using an Olympus CX40 fluorescence microscope mounted with a DS-5Mc charge-coupled device camera (Nikon). 

As a control, periplasmic FlaB was also investigated using unfixed as well as fixed spirochetes as described previously [39].

## 3. Results

### 3.1. CRASP-4 Interacts with Human Complement Regulators

**Figure 1:**
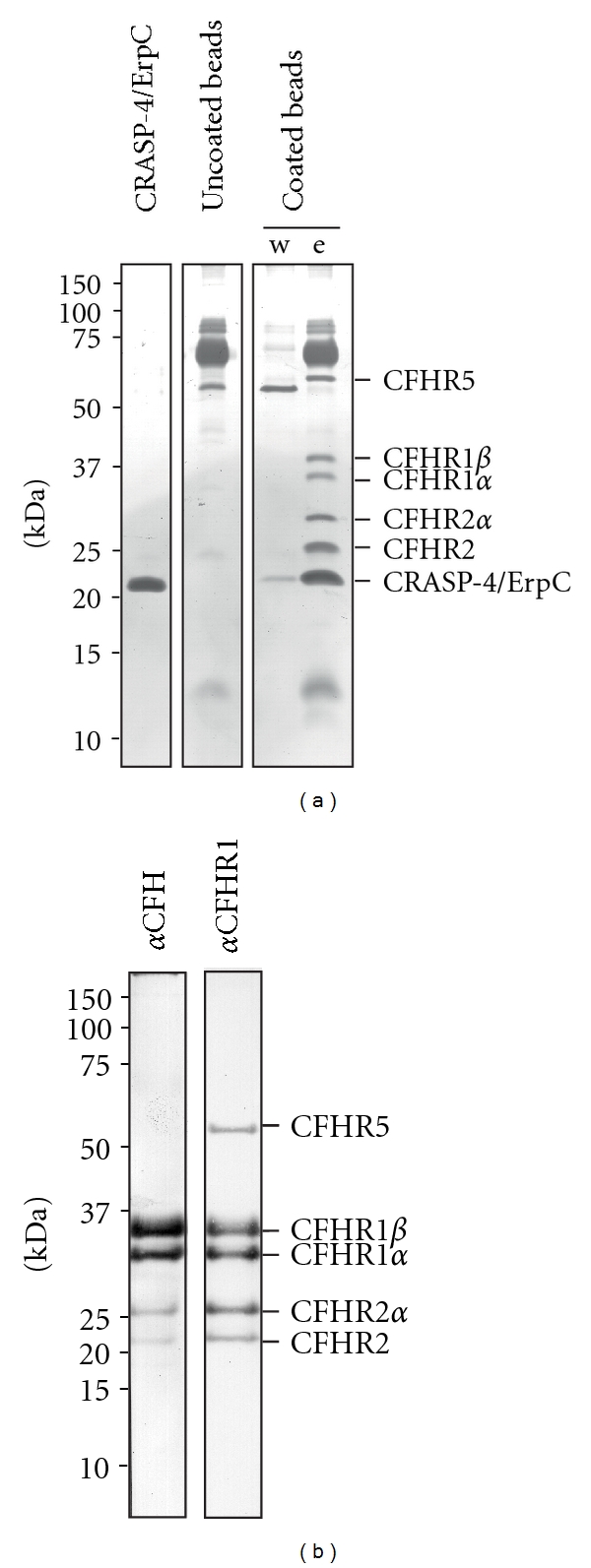
Identification of serum proteins that bind to recombinant CRASP-4. Recombinant, polyhistidine-tagged CRASP-4 was immobilized onto magnetic beads and incubated with NHS. Uncoated beads were also treated under the same conditions and used as a control to identify nonspecific binding of serum proteins. After extensive washing, bound proteins were eluted with 100mM glycine-HCl (pH 2.0) and the eluate fractions were separated by SDS-PAGE under nonreducing conditions. (a) Silver stain of a gel loaded with purified polyhistidine-tagged CRASP-4 (1 *μ*g), eluate fraction of the uncoated beads, and the final wash and eluate fraction of CRASP-4-coated beads. (b) Western blot analysis of the eluate fraction of CRASP-4-coated beads using a polyclonal anti-CFH or a polyclonal anti-CFHR1 antiserum. Mobilities of molecular mass standards are indicated to the left.

To identify serum components that bind to CRASP-4, the purified his-tagged protein was immobilized on magnetic beads. Following incubation with NHS, beads were extensively washed and bound serum proteins along with CRASP-4 were eluted. The eluate fraction of the CRASP-4-coated beads and eluate fraction of uncoated beads as well as the final wash were separated by SDS-PAGE and analyzed by silver staining (Figure 1(a)). A bulk of proteins in the 60 to 80 kDa range was detected in the eluate fractions of CRASP-4-coated beads and also in the eluate fraction of uncoated beads. In the eluate fraction of uncoated beads and in the final wash fraction but not in the eluate fraction of CRASP-4-coated beads, a 55 kDa protein was found. In contrast, proteins with apparent molecular masses of 60, 38, 35, 29, 25 and 22 kDa were detected only in the eluate fraction of CRASP-4-coated beads. 

To identify the serum proteins bound to the recombinant CRASP-4 protein, Western blot analysis was performed using specific antisera. All serum proteins bound to CRASP-4 react with the polyclonal anti-CFHR1 antiserum that recognizes the two different glycosylated forms of CFHR1 (CFHR1*α* and CFHR1*β*) and CFHR2 (CFHR2, and CFHR2*α*) as well as CFHR5 (Figure 1(b)). By using a polyclonal anti-CFH antiserum, four signals corresponding to CFHR1*α* (35 kDa) and CFHR1*β* (32 kDa) and CFHR2 (25 kDa) and CFHR2*α* (22 kDa) could be detected. The 150 kDa CFH protein was not detected by either silver staining or Western blot, indicating that CRASP-4 did not bind CFH under these experimental conditions. The protein with an apparent mass of 22 kDa represents CRASP-4. Taken together, CRASP-4 binds to human CFH protein family members, including CFHR1, CFHR2, and CFHR5.

**Figure 2:**
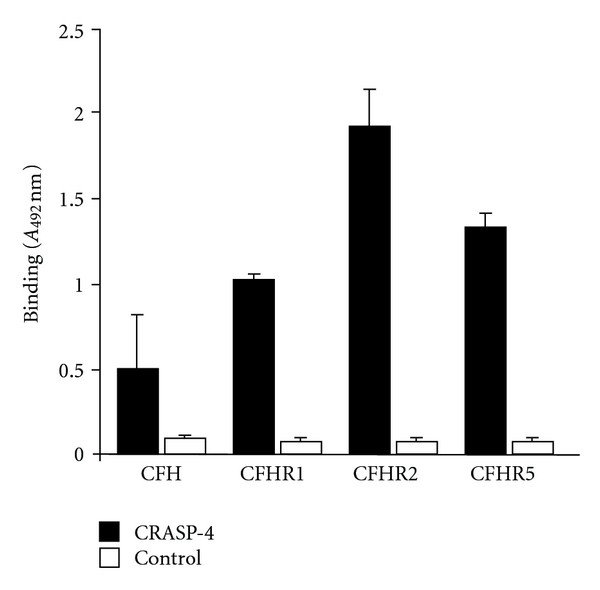
CRASP-4 binds distinct complement proteins. Binding of equimolar amounts of CFH, CFHR1, CFHR2, and CFHR5 (33 *μ*M) to immobilized CRASP-4 (5 *μ*g/mL) was analyzed by ELISA. Bound CFH or CFHR proteins were detected with either goat CFH polyclonal antiserum or mouse CFHR1 monoclonal antiserum (JHD 7.10), which reacts with all three CFHRs. Data represent the means and standard errors from three separate experiments.

Next, binding of recombinant CRASP-4 to each of the three identified human serum proteins was analyzed by ELISA (Figure 2). CRASP-4 was immobilized onto a microtiter plate and binding of purified recombinant CFHR1, CFHR2, CFHR5 and serum-purified CFH was assayed. All three CFHR proteins, that is, CFHR1, CFHR2, and CFHR5 as well as CFH bound to the immobilized CRASP-4 protein, with the greatest apparent affinity being for CFHR2.

### 3.2. Generation of a CRASP-4-Expressing *B. garinii* Strain

**Figure 3:**
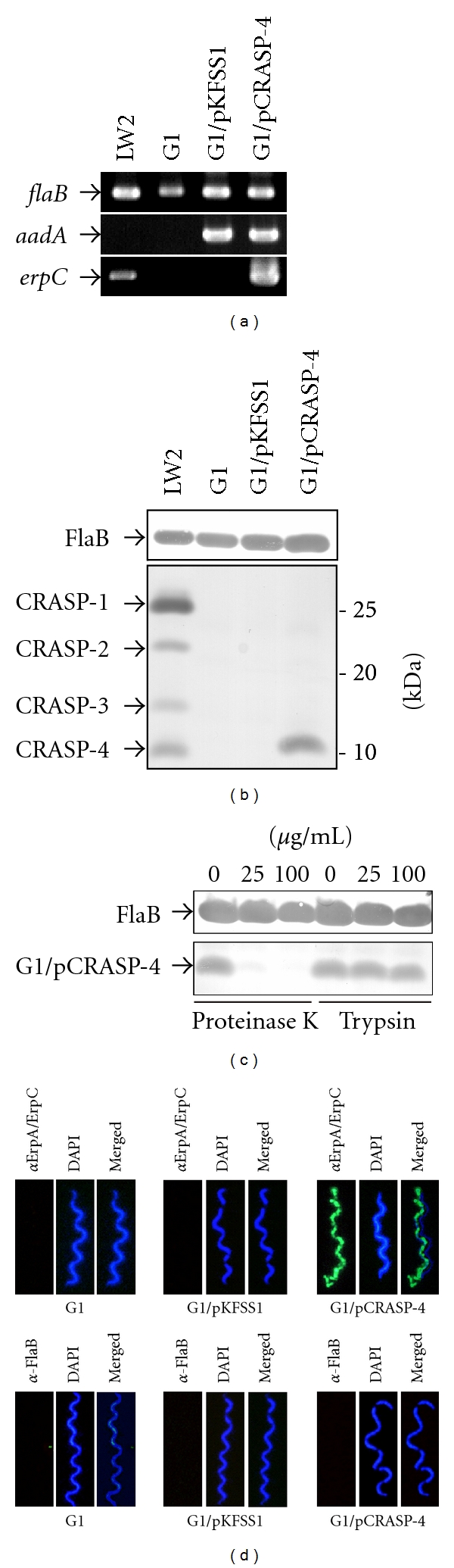
Characterization of *B. garinii* G1 producing CRASP-4. (a) *B. garinii *G1 and transformed strains G1/pKFSS1 and G1/pCRASP-4 were characterized by PCR amplification using *flaB-*, *aadA-*, and *erpC*-specific primers, as listed in Table 1. (b) Synthesis of CRASP-4 by transformed G1 was assessed using ligand affinity blotting. Whole cell lysates (15 *μ*g each) of G1, G1/pKFSS1 and G1/pCRASP-4 were separated by SDS-PAGE, and transferred to nitrocellulose. After incubation with NHS, binding of CFH to CRASP-4 was identified using a polyclonal antiserum. A monoclonal antibody, L41 1C11, specific for the flagellin protein FlaB, was applied to show equal loading of borrelial lysates. (c) Surface localization of CRASP-4 in transformed G1 cells. Spirochetes were incubated with or without proteinase K or trypsin, respectively, then lysed by sonication, and total proteins were separated by SDS-PAGE. CRASP-4 was identified by ligand affinity analysis as described above. Flagellin (FlaB) was detected with MAb L41 1C11 (dilution 1/1000) by Western blotting. (d) Demonstration of surface expression of CRASP-4 by transformed *B. garinii* G1, by indirect immunofluoresecence microscopy of intact borrelial cells. Spirochetes were incubated with rabbit polyclonal anti-ErpA/ErpC antiserum before fixation. Periplasmic FlaB, used as control, was detected by mAb L41 1C11 using fixed and unfixed cells. For counterstaining, the DNA-binding dye DAPI was used to identify all bacteria. Slides were visualized at a magnification of ×1,000 using an Olympus CX40 fluorescence microscope mounted with a DS-5Mc charge-coupled device camera (Nikon).

Depending on the genetic composition, all serum-resistant *B. burgdorferi* isolates analyzed to date express at least two distinct CRASP molecules. In order to assess the contribution of an individual CRASP molecule in mediating complement resistance, the serum-sensitive *B. garinii* strain G1 (does not express any of these CRASP proteins during laboratory cultivation) was chosen for functional analyses of the CRASP-4 protein [34]. *B. garinii *G1 was transformed with the plasmid pCRASP-4, which harbors the entire CRASP-4 encoding *erpC* gene under the control of its native promotor, and with the empty shuttle vector pKFSS1. Transformants selected by the microdilution method were confirmed by PCR amplification of the CRASP-4 encoding *erpC* and the streptomycin resistance* aadA* gene (Figure 3(a)). Strain G1/pCRASP-4 yielded an amplicon corresponding to *erpC*, whereas the control strains G1 and G1/pKFSS1 did not. The *aadA *gene of the shuttle vectors was detected in the transformed cells, but not in the wild-type strain G1. Production of CRASP-4 in* B. garinii* G1 was verified through analysis of cell lysates from the CRASP-4 expressing cells and the nonexpressing control strains G1 and G1/pKFSS1 (Figure 3(b)).

### 3.3. Surface Exposure of CRASP-4 in *B. garinii* G1/pCRASP-4

CRASP-4 and other members of the Erp paralogous protein family are surface exposed proteins [44]. To confirm surface-exposure of these proteins in transformed *B. garinii*, intact spirochetes were treated with proteinase K and trypsin, followed by ligand affinity blotting of borrelial lysates (Figure 3(c)). Analyses of protease-treated cells revealed that CRASP-4 was highly susceptible to digestion by proteinase K but not trypsin, as previously described for the native protein [44]. Surface localization of CRASP-4 was also examined by immunofluorescence microscopy using live bacteria and polyclonal antibodies specific for CRASP-4 [44]. To avoid damage to the fragile borrelial outer membrane, intact bacteria were incubated with antibodies before fixation onto glass slides and sealed with mounting medium containing the DNA-binding dye DAPI. As shown in Figure 3(d), CRASP-4 positive cells showed a strong fluorescent staining, thus indicating that CRASP-4 was localized on the outer membrane. Integrity of the fragile borrelial outer membrane was confirmed by the lack of binding of antibodies directed against the periplasmic flagellar protein FlaB (Figure 3(d)). Control strains G1 or G1/pKFSS1 did not display fluorescence reactivity with the CRASP-4 antiserum.

### 3.4. Binding of Human Serum Proteins by *B. garinii* G1/pCRASP-4

**Figure 4:**
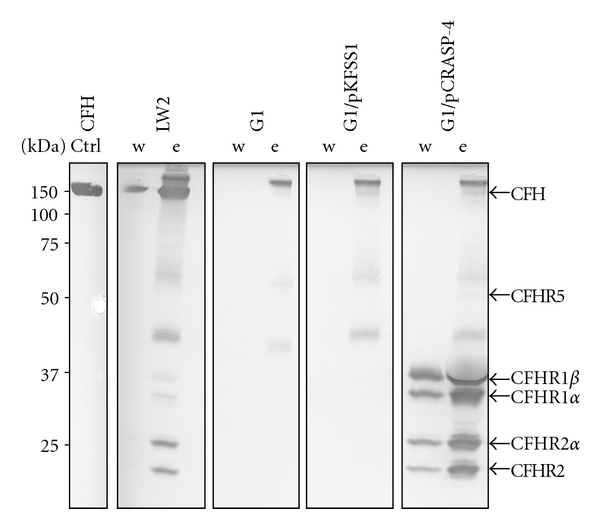
Binding of serum molecules by *B. garinii *transformants. *B. garinii* strains G1, G1/pKFSS1, and G1/pCRASP-4 and *B. burgdorferi* strain LW2 (used as control) were incubated in NHS plus EDTA to prevent complement activation and washed extensively, and bound proteins were eluted using 0.1 M glycine (pH 2.0). Both the last wash (w) and the eluate (e) fractions obtained from each strain were separated by SDS-PAGE and transferred to nitrocellulose. As an additional control purified CFH (1 *μ*g) was also applied. Membranes were probed with a polyclonal anti-FHR1 antiserum which recognizes CFH, CFHR1, CFHR2, and CFHR5. Mobilities of molecular mass standards are shown to the left of the panels.

Having demonstrated binding of CFHR1, CFHR2 and CFHR5 to recombinant CRASP-4, we next examined whether live G1/pCRASP-4 cells also bind the human complement regulators. To this end, serum-resistant *B. burgdorferi* LW2 (positive control), serum-sensitive *B. garinii* G1 (negative control), and transformants G1/pKFSS1 and G1/pCRASP-4 were incubated in NHS supplemented with EDTA (to prevent complement activation). After serum incubation, the final wash and elute fractions were separated by SDS-PAGE and subjected to Western blotting with a polyclonal antiserum that recognizes CFH and the CFH-related proteins CFHR1, CFHR1*α*, CFHR1*β*, CFHR2, CFHR2*α*, and CFHR5 (Figure 4). Serum-resistant* B. burgdorferi* LW2 bound CFH, CFHR1*α*, CFHR1*β*, CFHR2, and CFHR2*α*. In contrast, wild-type strain G1 and transformant G1/pKFSS1 did not bind CFH or any CFH-related proteins. Four prominent bands with apparent masses of 35, 32, 25 and 22 kDa were detected in the last wash and the eluate fraction of G1/pCRASP-4. Based on their mobilities, the 35 and 32 kDa proteins most likely correspond to the two glycosylated forms CFHR1*α* and CFHR1*β.*  The 25 and 22 kDa bands were probably the nonglycosylated and the glycosylated forms of CFHR2. A barely visible band with an apparent molecular mass of 55 kDa could only be detected in the eluate fraction of G1/pCRASP-4, which was probably CFHR5. The bands with molecular masses of 40, 60 and >250 kDa seen in the eluate fractions of all strains represent unspecific binding of the antiserum. There was not any indication of binding the 150 kDa CFH protein. Taken together, CRASP-4 produced on the surface of live *Borreliae* strongly binds the human serum proteins CFHR1 and CFHR2, lesser amounts CFHR5 but no detectable CFH.

### 3.5. Serum Susceptibility of *B. garinii* Producing Surface-Localized CRASP-4

**Figure 5:**
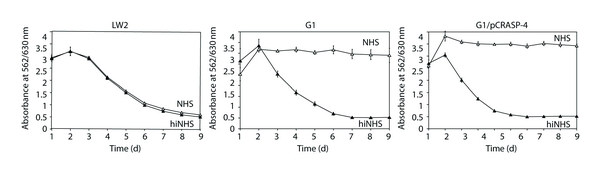
Serum susceptibility of transformed *B. garinii *G1. A growth inhibition assay was used to investigate susceptibility to human serum of *B. burgdorferi *strain LW2 and *B. garinii* strains G1 and G1/pCRASP-4. Spirochetes were incubated in either 50% NHS (open triangles) or 50% heat-inactivated NHS (filled triangles) over a cultivation period of 9 days at 33°C, respectively. Color changes were monitored by measurement of the absorbance at 562/630 nm. All experiments were performed three times during which each test was done at least in triplicate with very similar results. For clarity only data from a representative experiment are shown. Error bars represent ± SD.

To define the roles of CFHRs and CRASP-4 in the complement resistance of *Borreliae*, a growth inhibition assay was used to examine the ability of transformant G1/pCRASP-4 to survive in the presence of complement active NHS (Figure 5). As expected, growth of *B. burgdorferi* LW2 included as control was unaffected, as indicated by a continuous decrease of the absorbance values (due to the colour change of the medium). In contrast, wild-type strain G1, G1/pKFSS1, and G1/pCRASP-4 survived in heat-inactivated NHS but not in native NHS. The failure of the CRASP-4 producing transformant to survive suggests that binding of CFHR1, CFHR2, and CFHR5 is not sufficient for mediating complement resistance. 

**Figure 6:**
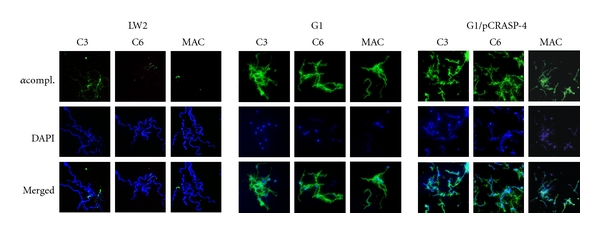
Deposition of complement components C3 and C6, and MAC on the surface of borrelial strains. Deposition of complement components on *B. burgdorferi* LW2 (control strain), *B. garinii* G1 and transformant G1/pCRASP-4 were detected by indirect immunofluorescence microscopy. Spirochetes were incubated with 25% NHS. Bound C3, C6, or MAC was detected using specific antibodies against each component plus appropriate Alexa-488-conjugated secondary antibodies. For visualization of intact spirochetes, the DNA-binding dye DAPI was used. Slides were visualized at a magnification of ×1,000 and the data were recorded via a DS-5Mc CCD camera (Nikon) mounted on an Olympus CX40 fluorescence microscope. Panels shown are representative of at least 20 microscope fields.

Next we examined deposition of complement activations products C3, C6 and the membrane attack complex (MAC) on the surface of the transformant G1/pCRASP-4, *B. burgdorferi* LW2, and *B. garinii* G1. Following incubation in NHS, the majority of cells of G1/pCRASP-4 and wild-type strain G1 displayed strong fluorescence, suggesting that large amounts of C3, C6, and MAC were deposited on the borrelial cell surface (Figure 6). In addition, extensive bleb formation, cell fragmentation, and lack of DAPI staining indicate that spirochetes were lysed. In contrast, bacteria incubated with heat-inactivated NHS did not show evidence of complement deposition (data not shown). 

Taken together, binding of CFHRs by CRASP-4 producing spirochetes does not sufficiently protect spirochetes from complement-mediated killing.

## 4. Discussion

To survive in different compartments and persistently infect their potential hosts, *Borreliae* have developed a variety of strategies that include overcoming destructive attacks by host complement. In particular, serum-resistant *B. burgdorferi*, *B. afzelii*, and *B. spielmanii* isolates bind the human fluid phase complement regulators CFH and FHL1 that allow spirochetes to finely regulate and inhibit complement activation on their cell surface [6, 7, 51]. In this study, we extend the characterization of molecular interaction of CFH/CFHR proteins and show that the infection-associated CRASP-4/ErpC protein of *B. burgdorferi* binds the host complement regulators CFHR1, CFHR2, and CFHR5, and to some extent CFH. However, CRASP-4 exposed to the outer surface of viable cells preferentially binds complement regulators CFHR1*α*, CFHR1*β*, CFHR2, and CFHR2*α*. 

CFHR1 and CFHR5, and likely also CFHR2, exhibit complement regulatory activities. Thus, recruitment of these host proteins may help spirochetes to control complement activation. In agreement with our earlier observations of the interaction of CFH with native CRASP-3 and CRASP-5, the data presented herein showed that CFHR1, CFHR2, and CFHR5 alone or in concert are not sufficient to control complement activation at the borrelial surface. CFHR1 and CFHR2 are major constituents of serum lipoprotein particles that also contain apolipoprotein A-I, lipopolysaccharide-binding protein, phospholipids, and fibrinogen [52, 53]. Thus, it could be speculated that Lyme disease *Borreliae* capture lipoprotein particles through CFHR1 and CFHR2 to allow adherence to host epithelial cells and tissues, as has been described for CFH-coated *Streptococcus pneumoniae* [54].

Interaction with CFH has previously been reported for CRASP-4/ErpC and other closely related Erp proteins, for example, OspE paralogs from *B. burgdorferi*, *B. afzelii*, *B. spielmanii*, *B. garinii*, *B. lusitaniae*, *B. turdi*, *B. tanukii*, and *B. japonica* [3, 6, 11, 12, 33, 35, 37, 38, 43, 51, 55–58]. Here we demonstrate that recombinant CRASP-4 bound CFH in ELISA or ligand affinity blot experiments using borrelial cell lysates (Figures 2 and 3(b)). However, binding of CFH could not be detected if CRASP-4 was coated onto magnetic particles or was expressed on the surface of transformed borrelial cells (Figures 1(a) and 1(b) and Figure 4). Previous studies using surface plasmon resonance revealed that CRASP-4 in comparison to CRASP-3 and CRASP-5 displayed strong affinity for CFHR1 and the lowest binding affinity to CFH, suggesting a preferential binding to the smaller CFHR molecules [38]. CRASP-3 and CRASP-5, when heterologously produced in *B. garinii* G1 or in a high-passaged mutant strain B313 (a derivative of type strain *B. burgdorferi* B31 that carries only one copy of the CRASP-5-encoding *erpA* gene), similarly did not bind CFH [31, 39]. However, we cannot completely exclude conformational changes of surface-exposed CRASP-4 due to misfolding in *B. garinii*. Furthermore, heterologous production of the two CFH/FHL1-binding CRASP proteins CspA or CspZ in *B. garinii* G1 did not influence their functional activity to interact with CFH and FHL1, which might also argue for correct folding of borrelial proteins in this model organism [42, and P. Kraiczy unpublished data]. Conceivably, the stronger affinity of CRASP-4 to CFHR1 and CFHR2 may also favor preferential binding of these molecules to borrelial cells (Figure 4), even though CFH is present in a 10-fold higher concentration in human plasma than both CFHRs [38, 59]. As demonstrated earlier and in the present study, the individual CFH/CFHR-binding CRASP proteins (recombinant or native) possess different relative affinities for CFH, CFHR1, CFHR2, and CFHR5 [38, 39, 59]. When expressed on borrelial surfaces, none of those CRASPs bound CFH, but they did show prominent binding to CFHR1 and CFHR2. Binding of CFHR5 was more pronounced for CRASP-3 as compared to CRASP-4 and CRASP-5. Collectively, all three CRASPs displayed the strongest affinities for CFHR2.

Apparently as a consequence of the inability of CFH to bind to the microbial surface, bacteria accumulated destructive complement activation products, that is, C3 and MAC, on their surfaces and were killed (Figures 5 and 6). Displacement of CFH by CFHR1 or CFHR2, which exhibits sequence identities of 89 and 61% to the C-terminal SCRs 19 and 20 of CFH, respectively, or improper binding of CFH to CRASP-4 by other yet unknown factors may have led to that phenomenon. Once complement is activated, it appears that the inhibitory activity of CFHR1 on the C5 convertase and the capacity of CFHR5 (although bound in minuscule amounts on the bacterial surface) to inactivate C3b can not completely impede formation and insertion of the MAC, in particular when large amounts of C3b and downstream effector complement components are deposited on the bacterial membrane (Figure 6). This points to a crucial role of human CFH and FHL1 in complement resistance of *Borreliae*.

A CspA-deficient *B. burgdorferi *strain that carries two native copies of the *erpA* gene did not survive in human serum, indicating that CRASP-5 alone cannot sufficiently protect Lyme disease *Borreliae* from complement-mediated killing [40, 41]. However, heterologous production of CRASP-3 and CRASP-5 in the same CspA-deficient strain significantly increased spirochetal survival in the presence of 20% human serum, suggesting that both proteins exhibit a synergistic effect on complement resistance [43]. However, mutant strain B313, which lacks CRASP-1, -2, and -3, but produces native CRASP-4 and -5, did not bind CFH and was highly susceptible to complement-mediated killing by 50% human serum (data not shown). Mutant strain B313 is a clonal mutant of B31 that lacks all that strain plasmids except cp32-1, cp32-2, cp32-3 cp32-4, cp26, and lp17 and therefore is unable to produce a variety of outer surface proteins, such as the major surface proteins OspA and OspB [60, 61]. The absence of a high number of outer surface proteins might influences the entire membrane composition and, thus, might effects the functional properties of these CFHR-binding CRASPs in the mutant strain B313. Conceivably, other proteins that are absent in B313 might serve as bystanders to promote optimal binding of the large CFH protein (which forms dimeric or oligomeric complexes in solution at physiological concentrations) to CRASP-3, CRASP-4, and CRASP-5.

Taken together, we identified complement proteins CFHR2 and CFHR5 as novel ligands for the infection-associated CRASP-4/ErpC protein of *B. burgdorferi*. CRASP-4 exposed to the borrelial surface preferentially binds CFHR1 and CFHR2 while binding of CFH and CFHR5 could only be detected under artificial experimental conditions. Although binding of CFHRs appears to be not necessary for complement resistance, the impact of these particular host proteins for immune evasion and pathogenesis of *Borreliae* warrants further investigations.

## Figures and Tables

**Table 1:** Oligonucleotides used in this study.

Oligonucleotide	Sequence (5′–3′)^a^	Use in this work
ErpC 5nc(+)	GTTGTATGTGTTTTGAAGCTTTTAGTAATGAGCAGGGC	Cloning in pKFSS1 and amplification of *erpC *
HindIII		
ErpC 3nc(−)	CGATCTCTCCTGTATTTTAAGCTTCTATTTTAAATTTTTCTTAAG	Cloning in pKFSS1 and amplification of of *erpC *
HindIII		
aadA + NdeI	CATATGAGGGAAGCGGTGATC	Amplification of *aadA* gene
aadR + AatII	GACGTCATTATTTGCCGACTACC	Amplification of *aadA* gene
Fla6	AACACACCAGCATCGCTTTCAGGGTCT	Amplification of *flaB* gene
Fla7	TATAGATTCAAGTCTATTTTGGAAAGCACCTA	Amplification of *flaB* gene

^a^Sequences of specific restriction endonuclease recognition sites are underlined.
